# Analysis of factors and pathways influencing patients’ level of worry about painless gastrointestinal endoscopy

**DOI:** 10.1097/MD.0000000000045070

**Published:** 2025-10-24

**Authors:** Lixiang Zhou, Liyu Lin, Xueyun Ye, Juan Feng, Yanting Wang, Yongtao Wu, Yunna Tang

**Affiliations:** a Department of Gastroenterology, Zhongshan Hospital Xiamen University, Xiamen, China; b Xiamen Gastroenterology Quality Control Center, Xiamen, China.

**Keywords:** factor analysis, mediating effect, painless gastrointestinal endoscopy, resilience, social support, worry

## Abstract

To investigate the level of worry in patients undergoing painless gastrointestinal endoscopy and to identify the factors and pathways that influence this worry. This cross-sectional study collected data between March and April 2024. A convenience sampling method was employed to select 477 patients scheduled for painless gastrointestinal endoscopy from a tertiary general hospital in Xiamen, China. The survey employed a self-administered general information questionnaire, along with validated scales measuring worry levels (WLs), psychological resilience (PR), and social support (SS). A multiple logistic regression analysis was used to investigate the factors influencing the level of worry. Model 4 of the PROCESS macro (v3.3) was used to analyze the mediating effects of PR on the relationship between SS and WLs. Worry scores were moderate (median score: 36, IQR: 25–47, on a 0–75 scale). Multivariate analysis revealed distinct predictors for different WLs. Rural residence was significantly associated with all three worry levels. Patients undergoing endoscopy for rechecking showed higher odds of mild worry, while those with fecal abnormalities and those enrolled in Urban Employee Basic Medical Insurance had increased odds of moderate worry. Lower monthly income (<¥ 4000) was associated with moderate worry. Younger age (<40 years) was the strongest predictor for severe worry. History of unsedated endoscopy, abdominal discomfort, and Urban Employee Basic Medical Insurance were also significant predictors for severe worry. Furthermore, PR mediated the relationship between SS and worry. Individualized communication and support plans should be developed based on different symptoms, medical histories, and specific patient characteristics. Particular attention should be paid to rural residents, younger patients (<40 years), those with lower incomes, patients experiencing abdominal discomfort or fecal abnormalities, and those with previous unsedated endoscopy experience. The type of medical insurance (Urban Employee Basic Medical Insurance) should also be considered as it was associated with both moderate and severe WLs.

## 1. Introduction

Gastrointestinal cancers pose a substantial threat to global health. A recent population-based analysis across 185 countries confirmed that gastric, esophageal, and colorectal cancers are among the leading causes of cancer-related mortality worldwide, with a significant and growing lifetime risk for individuals globally.^[1]^ China notably bears a particularly high burden, contributing to over 6,00,000 annual deaths from these cancers.^[2]^ The prognosis of gastrointestinal cancers is strongly stage-dependent, and early detection markedly improves survival outcomes compared with advanced disease.

The critical role of gastrointestinal endoscopy – a primary modality and the most effective test for early detection – has been endorsed by major international gastroenterological societies.^[3]^ Endoscopy allows for direct visualization and screening of precancerous lesions, and its efficacy in significantly reducing cancer-specific mortality has been demonstrated in large-scale randomized trials and organized national screening programs.^[4,5]^ However, the current population coverage of gastrointestinal endoscopy screening remains suboptimal. Therefore, it is imperative to improve the screening rate of gastrointestinal endoscopy.

Although gastrointestinal endoscopy is a well-established and relatively safe diagnostic and therapeutic technique, the adoption of painless procedures has alleviated patient discomfort; however, it remains an invasive intervention. Consequently, it can inflict considerable pain, and occasional incidents of anesthesia-related complications may lead to bleeding, perforation, and infection.^[6]^ Indeed, sedation itself carries inherent risks, a fact that is formally recognized and detailed in clinical guidelines worldwide.^[7]^ Furthermore, the high cost of painless endoscopy can impose a financial burden on patients. Consequently, despite the profound importance of this procedure, worries about undergoing painless gastrointestinal endoscopy remain widespread.^[8]^

Most existing studies in Western populations have focused on fear or anxiety as barriers to endoscopic screening. Qualitative research, for example, has shown that fear of pain and procedural discomfort is a key factor preventing attendance at colonoscopy.^[9]^ However, such fear typically reflects an immediate emotional response to perceived threat. In contrast, many patients experience worry – a future-oriented cognitive state characterized by persistent negative anticipation – well before the procedure takes place. This distinction is important, as worry may represent a more relevant psychological barrier to compliance with painless gastrointestinal endoscopy than fear or anxiety alone. In the limited research on cancer worry, higher levels of worry have been associated with greater intention to undergo colonoscopy; the strength of this association appears to depend on how worry is measured (e.g., intensity vs frequency).^[10,11]^ Together, these findings highlight the need to specifically examine worry as a distinct construct in endoscopic contexts.

The characteristics of worry encompass negative future-oriented thoughts, diminished attentional control, heightened focus on adverse information, and ambiguous negative interpretations.^[12–14]^ Worry is recognized as a primary symptom of generalized anxiety disorder. Yu et al determined that patients’ perceptions of sedation for gastrointestinal endoscopy were dominated by worry rather than anxiety.^[15]^ To assess patient concern regarding painless gastrointestinal endoscopy, the research team developed a pertinent scale in 2023. Gastrointestinal endoscopy acts as a stressor, often eliciting psychological concerns in patients, which, if unaddressed, can significantly impede their compliance and cooperation during the procedure. While existing research has primarily concentrated on patient anxiety, the determinants of worry in painless gastrointestinal endoscopy remain largely unexplored. Addressing this gap is essential for enhancing patient compliance and improving endoscopic tolerance.

Psychological resilience (PR) and social support (SS) play crucial roles in regulating individuals’ emotional states and in stress management. PR is defined as an individual’s capacity to adapt to and effectively manage adversity, trauma, pain, or tragedy.^[16]^ Robust PR enables individuals to maintain a healthy and stable psychological state.^[17]^ SS serves as a vital protective factor that mitigates emotional distress, enabling individuals to preserve their identity and receive emotional support through extensive social interactions. According to the classical buffering hypothesis, SS functions to alleviate the adverse impacts of stress on mental health.^[18]^ Concerning the relationship between worry levels (WLs), SS, and PR in patients undergoing painless gastrointestinal endoscopy, research remains nascent, with unclear mechanisms and pathways. Consequently, this study aimed to analyze the mediating effects of PR on the relationship between SS and WLs by establishing a mediation model and exploring the pathway of its influence.

This study aimed to explore the factors influencing patients’ WLs regarding painless gastrointestinal endoscopy and to analyze the pathways of impact, considering the mediating effects of PR and SS. The research will significantly enhance our comprehension of patients’ emotional experiences during painless gastrointestinal endoscopy, offer enhanced intervention strategies for clinical practice, and provide novel perspectives and insights for ongoing research and practice in related fields.

## 2. Materials and methods

### 2.1. Design

This cross-sectional study, conducted at a tertiary general hospital in Xiamen, China from March 1 to April 1, 2024, adhered to the Strengthening the Reporting of Observational Studies in Epidemiology (STROBE) guidelines (Appendix 1, Supplemental Digital Content, https://links.lww.com/MD/Q435).

### 2.2. Participants and sample

A convenience sampling method was employed at a tertiary general hospital in Xiamen, China. Inclusion criteria included individuals aged 18 years or older; outpatients or medical checkup patients who had scheduled an appointment for painless gastrointestinal endoscopy. Exclusion criteria comprised participants unable to or unwilling to independently complete the scale; patients scheduled for procedures such as polyp treatment, esophageal foreign body removal, or other complex gastrointestinal endoscopies; and patients diagnosed with well-defined gastrointestinal malignancies. All participants were fluent in Chinese, comprehended the questionnaire, and provided written informed consent. The sample size was determined using the Kendall M sample size formula, requiring 5 to 10 times the number of variables. This calculation was based on the 59 items across all scales used in the study. After accounting for 10% invalid questionnaires, the optimally calculated sample size ranged from 325 to 649. A total of 477 patients participated in the study.

### 2.3. Measures

#### 2.3.1. Demographic characteristics

A self-administered questionnaire was developed to gather sociodemographic information and details on disease-related conditions. The general information section of the questionnaire encompassed gender, age, marital status, height, weight, educational level, type of residence, home location, occupation, per capita monthly household income, method of paying for medical care, fertility status, smoking habits, and alcohol consumption. Disease-related information collected included past medical history, family history of digestive system cancer, reasons for consultation, previous experiences with general anesthesia and general gastrointestinal endoscopy, previous experience with painless gastrointestinal endoscopy, and the tests conducted during the current visit.

#### 2.3.2. Painless gastrointestinal endoscopy worry level

The gastrointestinal endoscopy with sedation worry level scale, developed by Yu et al, evaluates the degree of concern among patients scheduled for comprehensive painless gastrointestinal endoscopy.^[15]^ The scale is organized into 4 dimensions: financial and time costs, sedation, examination, and psychological factors, each represented by several items totaling fifteen. It utilizes a 5-point Likert scale, with responses ranging from 1 (“not at all worried”) to 5 (“extremely worried”), resulting in a total score range of 0 to 75. Elevated scores denote higher levels of concern, with scoring categories defined as follows: 0 to 18 for minimal concern, 19 to 37 for mild concern, 38 to 56 for moderate concern, and 57 to 75 for severe concern. The scale demonstrates high reliability, as evidenced by a Cronbach’s alpha coefficient of 0.959.

#### 2.3.3. Psychological resilience

The 10-item Connor–Davidson Resilience Scale, derived from the 25-item psychological resilience scale developed by Campbell-Sills and Stein^[19]^, functions as a tool for assessing individuals’ levels of PR in response to stress. Consisting of 2 dimensions, resilience (defined as the capacity to confront change and adversity) and strength (defined as the ability to exert maximum effort in adverse circumstances), each item on the scale is assessed using a 5-point Likert scale, with responses ranging from 0 (never) to 4 (almost always). The Chinese version of the scale, translated and widely utilized by Wang et al, is tailored for caregivers and patients coping with acute and chronic illnesses, exhibiting a Cronbach’s alpha coefficient of 0.910.

#### 2.3.4. Social support

The multidimensional scale of perceived social support, originally developed by Zimet et al in 1990, was introduced and subsequently revised in China by scholar Jiang Qianjin.^[20]^ It serves as a measure for assessing individuals’ self-perceived SS across various dimensions, such as family support (items 3, 4, 8, 11), friend support (items 6, 7, 9, 12), and other support types (items 1, 2, 5, 10). The scale comprises 12 items and employs a 7-point Likert scale, with responses ranging from “strongly disagree” (1 point) to “strongly agree” (7 points). Higher scores indicate a heightened subjective perception of SS by the individual. The scale has been validated among hospitalized patients in China, demonstrating strong reliability and validity, as evidenced by a Cronbach’s alpha coefficient of 0.922.^[21]^

### 2.4. Data collection

The survey was distributed in both electronic and paper formats. Survey enumerators, who had completed standardized training, provided uniform instructions to explain the survey’s purpose, significance, and completion methods to the patients. After obtaining informed consent, respondents were offered the option to immediately complete the questionnaire on-site by scanning the QR code for the electronic version. The electronic questionnaire, developed via the "Wen Juan Xing" platform, made all responses mandatory to ensure completeness. Moreover, restrictions were implemented on each IP address to prevent duplicate submissions. Paper questionnaires were completed and collected on-site, undergoing thorough inspections for omissions to preserve data integrity and validity. Each questionnaire was subsequently subjected to a meticulous review, with exclusion criteria targeting those completed in under 90 seconds or those with uniform responses across all items. A total of 477 questionnaires were collected, with 474 deemed valid, achieving an effective response rate of 99.37% and ensuring a sufficient sample size.

### 2.5. Data analysis

The data collected were carefully checked for integrity and consistency before being cleaned for analysis. Statistical analyses were performed using SPSS version 26.0 (IBM Corp., Armonk ). Continuous variables were presented as medians and interquartile ranges, whereas categorical data were presented as frequencies and percentages. The Chi-square test was used to assess differences in the distribution of worry levels (categorical grades) regarding painless gastrointestinal endoscopy between the general and disease-specific datasets. Variables showing significant statistical differences in univariate analyses were included in a multivariate logistic regression analysis. Associations between levels of worry about painless gastrointestinal endoscopy, SS and PR were examined using Spearman’s correlation analysis. The mediating function of PR between SS and worry about painless gastrointestinal endoscopy was assessed using model 4 of the SPSS Process macro v3.3, with worry about endoscopy as the dependent variable (*Y*), SS as the independent variable (*X*), and PR as the mediator (*M*), using the bootstrap technique to assess mediation effects. A *P*-value below .05 was considered statistically significant.

### 2.6. Ethical considerations

The study was approved by the Ethics Committee of Zhongshan Hospital Affiliated to Xiamen University (2024-068). Participation was voluntary, and written informed consent was obtained from all participants. The purpose of the study was explained to participants, along with options for anonymity, confidentiality, and withdrawal, prior to administering the survey. No compensation or rewards were provided for participation in the study.

## 3. Results

### 3.1. Painless gastrointestinal endoscopy worry level score

The overall median score of the gastrointestinal endoscopy with sedation worry level scale was 36 (IQR 25–47), indicating a moderate level of worry among patients (Table 1). When comparing the median WL per item (to account for the different number of items in each dimension), sedation-related concerns were the highest, with a median score of 2.67 per item (IQR 1.67–3.33), followed by examination-related worries [2.50 (IQR 1.67–3.17)] and psychological concerns [2.33 (IQR 1.67–3.33)]. In contrast, financial and time cost worries were the lowest, with a median of 2.00 per item (IQR 1.00–3.00). These findings suggest that patients were particularly apprehensive about the anesthesia process itself, while economic considerations were comparatively less prominent.

**Table 1:** Painless gastrointestinal endoscopy worry level scores (n = 474).

Variable	Scores median (P25, P75)	Average score for each entry median (P25, P75)
Financial and time costs	6.00 (3.00, 9.00)	2.00 (1.00, 3.00)
Sedation	8.00 (5.00, 10.00)	2.67 (1.67, 3.33)
Examination	15.00 (10.00, 19.00)	2.50 (1.67, 3.17)
Psychology	7.00 (5.00, 10.00)	2.33 (1.67, 3.33)
Gastrointestinal endoscopy with sedation worry level scale	36.00 (25.00, 47.00)	2.40 (1.67, 3.13)

### 3.2. Univariate analysis of the level of worry about anesthesia for gastroenteroscopy

Univariate analysis revealed that sociodemographic characteristics such as age, education, living status, family location, and monthly household income were significantly associated with the level of worry about painless gastrointestinal endoscopy (all *P* < .05). Although younger patients (<40 years) showed differences in worry levels, those with higher household income (>¥ 8000/mo) reported lower levels of worry, whereas patients living in rural areas or with lower education levels tended to experience greater worry. Living alone was also related to increased worry. Medical payment method and fertility status were further associated with the outcome, with patients covered by Urban and Rural Resident Basic Medical Insurance and those without children showing higher levels of worry compared to their counterparts.

Lifestyle and health-related factors demonstrated notable associations as well. Individuals with a history of smoking or drinking, those with antecedent medical conditions, and patients with a family history of digestive system cancer were more likely to report elevated worry (all *P* < .05).

Regarding clinical motivations, patients undergoing endoscopy due to fecal abnormalities, follow-up examinations, high-risk screening, or routine medical checkups expressed greater worry compared with those undergoing the procedure for abdominal discomfort (all *P *< .05). Previous experience with anesthetic-free endoscopy was also linked to increased worry. Finally, the nature of the planned examination influenced patients’ WLs: those scheduled for gastroscopy or colonoscopy reported more severe worry than those undergoing combined gastrointestinal endoscopy. Detailed statistical values are presented in Table 2.

**Table 2:** Study of Painless gastrointestinal endoscopy worry level scores based on different characteristics.

Variable	Categories	n	Minimal level of worry	Mild worry	Moderate worry	Severe worry	χ^2^	*P*
Age (yr)	<40	308	47	95	118	48	27.530	<.001
	40–59	129	30	61	33	5		
	≥60	37	7	11	16	3		
Educational level	Junior high school and below	75	13	22	26	14	17.380	.008
	High school or junior college	96	9	30	40	17		
	College and above	303	62	115	101	25		
Live alone	Yes	62	14	13	21	14	12.106	.007
	No	412	70	154	146	42		
Family location	city	272	67	110	74	21	51.145	<.001
	County or township	114	13	29	47	25		
	rural	88	4	28	46	10		
Monthly per capita household income	<¥4000	105	11	27	53	14	61.185	<.001
	¥4000–¥8000	210	31	59	85	35		
	>¥8000	159	42	81	29	7		
Medical payment methods	Urban and Rural Resident Basic Medical Insurance	173	23	40	81	29	30.385	<.001
	Urban Employee Basic Medical Insurance	301	61	127	86	27		
Fertility	No children	176	25	53	76	22	18.645	.005
	1 child	181	30	63	62	26		
	≥2 children	117	29	51	29	8		
Smoking history	Yes	189	16	56	83	34	34.869	<.001
	No	285	68	111	84	22		
Drinking history	Yes	281	39	96	105	41	11.369	.010
	No	193	45	71	62	15		
Antecedent history	Yes	106	17	23	41	25	23.788	<.001
	No	368	67	144	126	31		
Family history of cancer of the digestive system	Yes	77	12	14	29	22	29.828	<.001
	No	397	72	153	138	34		
Reasons for planning a gastrointestinal endoscopy								
Abdominal discomfort	Yes	191	29	58	73	31	9.404	.024
	No	283	55	109	94	25		
Fecal abnormality	Yes	153	15	42	74	22	24.194	<.001
	No	321	69	125	93	34		
Rechecking	Yes	119	8	39	53	19	17.343	.001
	No	355	76	128	114	37		
Screening of high-risk groups	Yes	92	4	20	52	16	35.112	<.001
	No	382	80	147	115	40		
Medical checkup	Yes	218	35	96	76	11	25.181	<.001
	No	256	49	71	91	45		
Previous experience of anesthetic-free gastrointestinal endoscopy	Yes	157	20	47	55	35	26.978	<.001
	No	317	64	120	112	21		
Examination items planned for this time	Gastroscopy	147	22	36	58	31	42.164	<.001
	Colonoscopy	79	6	26	35	12		
	Gastrointestinal endoscopy	248	56	105	74	13		

### 3.3. Multiple logistic regression analysis of factors affecting gastroenterology worries

Based on the multivariate logistic regression analysis of variables that showed significance in univariate analyses (Table 3), several independent predictors were identified for different levels of procedural worry.

**Table 3:** Multiple logistic regression analysis of level of worry about painless gastrointestinal endoscopy.

Categories	Mild worry*			Moderate worry*			Severe worry*		
	*P*	OR	95% CI	*P*	OR	95% CI	*P*	OR	95% CI
Age (vs >60)									
<40 yr	.375	1.808	0.489–6.69	.268	2.117	0.562–7.977	.005	19.285	2.395–155.298
40–60 yr	.364	1.782	0.512–6.197	.838	1.143	0.319–4.100	.383	2.494	0.32–19.411
Educational level (vs Junior high school and below)									
College and above	.992	1.005	0.374–2.702	.406	1.565	0.545–4.494	.549	0.654	0.163–2.622
High school or junior college	.481	1.515	0.477–4.813	.734	1.232	0.37–4.101	.621	0.682	0.15–3.100
Live alone (vs no)	.109	0.454	0.172–1.193	.059	0.397	0.152–1.036	.739	0.819	0.253–2.651
Family location (vs city)									
Rural	.008	4.977	1.524–16.257	.002	6.638	2.033–21.676	.020	5.592	1.316–23.769
County or township	.524	1.310	0.571–3.009	.434	1.390	0.609–3.172	.131	2.210	0.79–6.183
Monthly per capita household income (vs >$8000)									
<¥4000	.900	0.940	0.358–2.468	.016	3.390	1.25–9.191	.217	2.471	0.588–10.381
¥4000–¥8000	.332	0.720	0.371–1.398	.06	2.023	0.971–4.217	.246	1.952	0.63–6.048
Medical payment methods (vs Urban and Rural Resident Basic Medical Insurance)									
Urban Employee Basic Medical Insurance	.309	0.692	0.341–1.406	.046	2.051	1.014–4.149	.028	2.850	1.118–7.268
Fertility (vs no children)									
1 child	.704	1.172	0.517–2.658	.755	1.143	0.494–2.643	.714	1.227	0.412–3.651
≥2 children	.609	0.800	0.339–1.884	.147	0.512	0.207–1.267	.263	1.771	0.651–4.820
Reasons for planning a gastrointestinal endoscopy									
Smoking history (vs no)	.509	1.280	0.615–2.663	.316	1.479	0.689–3.178	.263	1.771	0.651–4.820
Drinking history (vs no)	.453	1.255	0.694–2.270	.240	1.468	0.774–2.784	.247	1.723	0.686–4.325
Antecedent history (vs no)	.579	0.805	0.374–1.733	.340	1.481	0.661–3.319	.093	2.555	0.855–7.634
Family history of cancer of the digestive system (vs no)	.564	0.764	0.305–1.909	.662	1.229	0.488–3.095	.072	2.850	0.910–8.930
Abdominal discomfort (vs no)	.251	1.479	0.758–2.885	.319	1.421	0.712–2.833	.015	3.159	1.249–7.991
Fecal abnormality (vs no)	.262	1.531	0.728–3.222	.008	2.765	1.309–5.839	.120	2.148	0.820–5.628
Rechecking (vs no)	018	3.022	1.206–7.574	.051	2.556	0.996–6.56	.124	2.427	0.785–7.509
Screening of high-risk groups (vs no)	.528	1.507	0.422–5.39	.098	2.799	0.828–9.46	.13	2.923	0.729–11.722
Medical checkup (vs no)	.003	2.775	1.421–5.418	.173	1.632	0.807–3.301	.346	0.611	0.219–1.704
Previous experience of anesthetic-free gastrointestinal endoscopy (vs no)	.876	1.054	0.544–2.043	.426	1.328	0.661–2.67	.004	3.6	1.488–8.71
Examination items planned for this time (vs gastrointestinal endoscopy)									
Gastroscopy	.878	0.947	0.472–1.900	.325	1.432	0.701–2.925	.068	2.494	0.935–6.653
Colonoscopy	.288	1.776	0.616–5.123	.382	1.626	0.547–4.831	.451	1.737	0.414–7.29

CI = confidence interval, OR = odds ratio.

* Minimum level of worry as the reference group.

For the mild worry category, patients residing in rural areas (odds ratio [OR] = 4.977, 95% confidence interval [CI]: 1.524–16.257, *P* = .008) and those undergoing endoscopy for rechecking (OR = 3.022, 95% CI: 1.206–7.574, *P* = .018) or physical examinations (OR = 2.775, 95% CI: 1.421–5.418, *P* = .003) showed significantly higher odds of classification in this group.

For the moderate worry category, significant predictors included: rural residence (OR = 6.638, 95% CI: 2.033–21.676, *P* = .002), monthly income < ¥ 4000 (OR = 3.390, 95% CI: 1.250–9.191, *P* = .016), stool abnormalities (OR = 2.765, 95% CI: 1.309–5.839, *P* = .008), and enrollment in Urban Employee Basic Medical Insurance (OR = 2.051, 95% CI: 1.014–4.149, *P* = .046).

For the severe worry category, the strongest predictors were: age < 40 years (OR = 19.285, 95% CI: 2.395–155.298, *P* = .005), rural residence (OR = 5.592, 95% CI: 1.316–23.769, *P* = .020), abdominal discomfort (OR = 3.159, 95% CI: 1.249–7.991, *P* = .015), history of unsedated endoscopy (OR = 3.600, 95% CI: 1.488–8.710, *P* = .004), and Urban Employee Basic Medical Insurance (OR = 2.850, 95% CI: 1.118–7.268, *P* = .028).

### 3.4. Correlations between levels of worry about painless gastrointestinal endoscopy and social support and psychological resilience

Spearman’s correlation analysis identified a robust positive correlation between PR and SS (*r* = 0.671, *P* < .001), alongside a significant negative correlation between resilience and WLs (*r* = −0.258, *P* < .001). Additionally, a notable negative correlation was observed between SS and WLs (*r* = −0.285, *P* < .001).

### 3.5. The mediating role of psychological resilience between social support and worry levels

**Figure 1.**
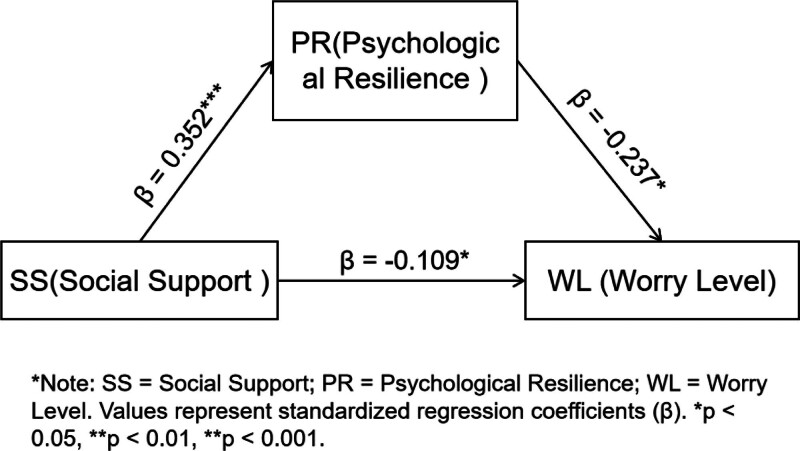
Mediating effects model of psychological resilience. The figure illustrates the mediating role of psychological resilience in the relationship between social support and worry level regarding painless gastrointestinal endoscopy. Values on the arrows represent standardized regression coefficients (β). SS assessed by the perceived social support scale (PSSS); PR assessed by the Connor–Davidson Resilience Scale-10 (CD-RISC-10); WL assessed by the Painless Gastrointestinal Endoscopy Worry Scale. Social support positively predicted psychological resilience (β = 0.352, *P* < .001), which in turn negatively predicted worry level (β = −0.237, *P* < .05). Social support also directly and negatively predicted worry level (β = −0.109, *P* < .05). Detailed total, direct, and indirect effects are reported in Tables 4 and 5. CD-RISC-10 = Connor–Davidson Resilience Scale-10, PR = psychological resilience, PSSS = perceived social support scale, SS = social support, WL = worry level.

The mediation model was established using WL as the dependent variable, SS as the independent variable, and PR as the mediator, as depicted in Figure 1. The analysis was conducted with model 4 of the SPSS Process v3.3 after standardizing all variable scores. Results demonstrated that SS negatively predicted WL (β = −0.193, *P* < .001). When PR was introduced as a mediator, SS still exerted a direct negative effect on WL (β = −0.109, *P* < .05). In addition, SS positively predicted PR (β = 0.352, *P* < .001), which in turn negatively predicted WL (β = −0.237, *P *< .05). Detailed results are provided in Table 4.

**Table 4:** Mediating model test results of psychological resilience between social support and worry levels.

Outcome variable	Predictive variable	Fitting index		Significance of coefficient	
		*R* ^2^	*F*	*t*	β
Worry level	Social support	0.0459	22.700	−4.765	−0.193**
Psychological resilience	Social support	0.423	345.511	18.588	0.352**
Worry level	Psychological resilience	0.0576	14.393	−2.419	−0.237*
	Social support			−2.066	−0.109*

* *P* < .05.

** *P* < .01.

**Table 5:** Total effect, direct effect and indirect effect of psychological resilience between social support and worry levels.

Item	Effect size	Standard error	95% confidence interval		Relative effect value
			Low	High	
Total effect	−0.193	0.043	−0.278	−0.109	
Indirect effect	−0.083	0.037	−0.155	−0.010	0.432
Direct effect	−0.109	0.058	−0.224	0.004	0.568

Bootstrap tests with 5000 resamples further confirmed the mediation effect, with 95% confidence intervals. The indirect effect of PR was −0.083 (95% CI: −0.155 to −0.010), while the direct effect of SS was −0.109 (95% CI: −0.224 to −0.004). This indicates that SS not only directly reduces worry about painless gastrointestinal endoscopy but also indirectly reduces worry by enhancing PR. The direct and indirect effects accounted for 56.8% and 43.2% of the total effect, respectively (see Table 5).

## 4. Discussion

The average level of worry was recorded at 48.71%, classified as moderate. A breakdown of the worry categories showed that 17.7% of patients experienced minimal worry, 35.2% mild worry, a further 35.2% moderate worry and 11.8% severe worry – figures that were generally lower than those reported in the study by Yu et al^[15]^ This variation can be attributed to the fact that the study by Yu et al included all gastrointestinal endoscopy patients, whereas this study focused only on patients without confirmed gastrointestinal malignancy. Further analysis revealed that the sedation dimension elicited the highest mean worry score, whereas financial and time costs elicited the lowest. This discrepancy is likely to be due to heightened concerns about the uncertainties and risks associated with anesthetic sedation. Conversely, the predictability of financial and time costs may explain the lower levels of worry about this dimension.^[22]^ In addition, the inherent risks of anesthesia, including loss of consciousness and reliance on medical staff for safety, may increase patient apprehension.^[23]^ A lack of understanding of anesthesia processes also contributes to increased worry in some patients.

These findings align with international literature. A systematic review from Europe and North America demonstrated that patients undergoing colonoscopy or sigmoidoscopy frequently report anticipatory concerns related to pain, sedation, embarrassment, and procedural risks.^[24]^ Such overlap underscores the universality of sedation-related worry across cultural contexts. Future interventions should therefore include structured pre-procedure education, flowchart-based demonstrations of sedation steps, public disclosure of anesthetist qualifications, and recovery management manuals to enhance understanding. Moreover, distraction-based interventions, such as audiovisual distraction, have been shown to significantly reduce anxiety and pain while improving satisfaction during endoscopy.^[25]^

It is important to identify the factors that influence the level of anxiety experienced by patients undergoing painless gastrointestinal endoscopy. Multinomial logistic regression revealed that variables such as rural residence, indication for endoscopy, monthly income, type of health insurance, age, and history of unsedated endoscopy were significant predictors of WL.

Compared with their urban counterparts, rural patients expressed greater concern about pain-free gastrointestinal endoscopy. Ma et al showed that people in rural areas were more likely to refuse gastroscopy.^[26]^ This reluctance may be due to economic constraints, limited medical information, and higher costs of painless gastrointestinal endoscopy, which together foster hesitation and worry.^[27,28]^ In Western countries with universal healthcare, cost concerns are less prominent, and discomfort or procedural anxiety tends to dominate as barriers. Therefore, policy measures in China should prioritize financial subsidies, investment in local health education, and outreach initiatives to strengthen trust.

The indication for endoscopy significantly stratified WLs. Patients attending for routine physical examination exhibited lower levels of worry, likely because they are typically asymptomatic and perceive the procedure as preventive. In contrast, patients presenting for reexamination showed a trend toward moderate worry (OR = 2.556, *P* = .051). This suggests that prior experience does not necessarily mitigate worry and may even exacerbate it, possibly because a referral for reexamination implies a known history of abnormalities, shifting fear from the unknown procedure to the potential findings.

In contrast, stool abnormalities may indicate underlying bowel disease. Uncertainty and fear of symptoms may elevate the patient’s level of worry, potentially categorizing them within the moderate worry group.^[29]^ For patients undergoing gastrointestinal endoscopy due to abdominal discomfort, this procedure is likely to induce a higher level of worry. Among our patients undergoing gastroscopy, the most common symptoms include dyspeptic issues, predominantly upper-middle abdominal pain, bloating, and acid reflux.^[30]^ Abdominal discomfort, often a pronounced and persistent symptom, may exacerbate worry. Healthcare professionals should therefore not only address the procedural aspects but also provide personalized communication strategies that clarify the diagnostic value of endoscopy, normalize the presence of functional symptoms, and reduce catastrophic interpretations. This patient-centered approach can improve understanding and cooperation, and ultimately optimize patients’ experience and outcomes.

Furthermore, the type of health insurance emerged as a significant predictor. Patients enrolled in the urban-rural resident insurance scheme had higher odds of moderate (OR = 2.051, *P* = .046) and severe worry (OR = 2.85, *P* = .028). This association may reflect socioeconomic disparities, as differences in reimbursement coverage and out-of-pocket costs can directly influence perceived financial burden and procedural anxiety.

Patients who had previously undergone general gastrointestinal endoscopy were more likely to be classified in the severe worry group. General gastrointestinal endoscopies are performed while patients are fully conscious, which may cause discomfort. Those with unpleasant past experiences may experience negative emotions, leading to apprehension about subsequent procedures.^[31]^ This pattern may reflect a “carry-over effect” of traumatic memory, whereby unpleasant experiences during conscious procedures amplify current worries even in the context of painless techniques. Medical teams should therefore strengthen pre-procedure counseling, emphasizing the fundamental differences between general and painless endoscopy, highlighting the use of modern sedation techniques, and reassuring patients about safety and comfort. In addition, testimonials from prior patients with positive experiences could be integrated into routine education to reduce fear.

A higher proportion of patients under 40 years of age were categorized in the painless gastrointestinal endoscopy “high worry” group compared to those over 40 years of age. A similar trend was observed in a Spanish study, where younger colonoscopy patients exhibited significantly higher levels of anxiety.^[32]^ Several reasons may explain this trend: younger individuals often face heavier family and career responsibilities, are more susceptible to social expectations, and are more exposed to online medical information, including negative reports, which may exacerbate worry.^[33]^ This suggests that worry is not only a medical issue but also intertwined with social and generational pressures. Younger adults may overestimate procedural risks due to fragmented or sensationalized online health information, while simultaneously underestimating their ability to cope. Targeted psychological interventions for this group could include digital health education via reliable platforms and peer-led support groups, addressing both cognitive distortions and emotional regulation.

Patients with lower household income were more likely to be moderately worried compared with higher-income patients. Low income may increase concerns about the high cost of endoscopy, as also identified as a major reason for refusal in the Ma study.^[26]^ Financial strain can contribute to family stress and reduce family support. This highlights the role of socioeconomic disparities, which remain particularly pronounced in China due to partial self-payment for painless endoscopy despite relatively low absolute medical costs. In contrast, in many Western countries with universal healthcare coverage, financial concerns are less salient, and procedural discomfort or embarrassment tends to be the primary barrier. These differences underscore the importance of tailoring policy interventions: in China, cost-reduction strategies and subsidies are paramount, while in Western systems, efforts may better focus on patient comfort and reducing stigma.

The mediation analysis indicated that PR exerted a partial mediating effect between SS and worry, accounting for 43.2% of the total effect. This suggests that SS can both directly and indirectly influence WLs through resilience. Individuals with higher resilience tend to exhibit more positive attitudes, enhanced coping skills, and improved emotional regulation, enabling them to adapt more quickly to new situations and reduce worry.^[34]^ The mediating role of resilience has also been observed in other cultural contexts, including Europe and the United States, suggesting its universal significance.^[35]^ SS not only provides direct emotional and material resources but also fosters resilience by reinforcing patients’ sense of competence and security. Strengthening family involvement, peer support, and community-based mental health resources may therefore serve as critical strategies to buffer procedural worry and improve patient outcomes.

### 4.1. Limitation

Our study also had several limitations. Firstly, owing to the restricted representativeness of the sample, only patients scheduled to undergo gastrointestinal endoscopy in a tertiary general hospital in Xiamen, China, were included. This could result in regional bias and compromise the broader applicability of the results. Expanding the scope and conducting a multicentre, large-sample survey would be advantageous in future studies. This study utilized a cross-sectional design, thereby precluding the observation of changes and developments in patients before and after undergoing gastrointestinal endoscopy. This limitation might have hindered a deeper understanding of patients’ concerns. Future research could involve a longitudinal study to observe the dynamic changes in patients’ WLs. Third, this study focused solely on the mediating role of PR between SS and WLs. Subsequent research could explore additional potential mediating variables, such as health literacy and coping styles, to better understand how PR influences the relationship between SS and WLs.

## 5. Conclusion

The present study revealed that worry about anesthesia-free gastrointestinal endoscopy ranged from moderate to severe, with significant variations according to patients’ residence, reason for undergoing the procedure, household income, age, and previous endoscopy experience. PR was identified as a mediator in the association between SS and patient worry. These findings suggest that the government and healthcare agencies should increase investment in rural healthcare resources, reduce the economic burden of painless gastrointestinal endoscopy, and strengthen SS systems. Clinicians are encouraged to provide comprehensive information and psychological support to improve patients’ health literacy, confidence, and understanding of the procedure. Tailored communication and support should be prioritized for rural residents, older adults, patients with prior endoscopy experience, and those with lower income. Future research should further examine the effectiveness of targeted interventions to reduce patient worry, thereby optimizing the clinical application of painless gastrointestinal endoscopy and improving overall patient experiences.

## Author contributions

**Data curation:** Liyu Lin, Juan Feng, Yanting Wang, Yongtao Wu, Yunna Tang.

**Investigation:** Lixiang Zhou, Liyu Lin, Xueyun Ye, Juan Feng, Yanting Wang, Yongtao Wu, Yunna Tang.

**Methodology:** Lixiang Zhou, Liyu Lin.

**Resources:** Lixiang Zhou.

**Validation:** Xueyun Ye.

**Writing – original draft:** Liyu Lin, Xueyun Ye.

## Supplementary Material


